# Mangosteen Pericarp Processing Technology to Create Economic Value and Reduce Biowaste

**DOI:** 10.3390/foods13142286

**Published:** 2024-07-20

**Authors:** Alisa Soontornwat, Thadchapong Pongsuttiyakorn, Samak Rakmae, Eakasit Sritham, Panmanas Sirisomboon, Umed Kumar Pun, Warawut Krusong, Pimpen Pornchaloempong

**Affiliations:** 1 Department of Food Engineering, School of Engineering, King Mongkut’s Institute of Technology Ladkrabang, Bangkok 10520, Thailand; alisa.peep@gmail.com (A.S.); tpsky.7@gmail.com (T.P.); samak.ra@kmitl.ac.th (S.R.); eakasit.sr@kmitl.ac.th (E.S.); 2 Department of Agricultural Engineering, School of Engineering, King Mongkut’s Institute of Technology Ladkrabang, Bangkok 10520, Thailand; panmanas.si@kmitl.ac.th (P.S.); umedpun@gmail.com (U.K.P.); 3 Division of Fermentation Technology, School of Food industry, King Mongkut’s Institute of Technology Ladkrabang, Bangkok 10520, Thailand; warawut.kr@kmitl.ac.th

**Keywords:** a mobile processing system, *α*-mangostin, mangosteen pericarps, extraction, zero waste, upcycle, valorization

## Abstract

This research comparatively investigates different mangosteen pericarp processing schemes. The experimental pericarp processing schemes were hot air drying (HAD; control), quick freezing/HAD (QF + HAD), slow freezing/HAD (SF + HAD), and slow freezing/freeze-drying (SF + FD). For freezing, the QF temperature was −38 °C for 2 h and that of SF was −25 °C for 2 weeks. For drying, the HAD temperature was 60 °C for 7 h. In the FD process, the primary and secondary temperatures were −20 °C and 50 °C for 48 h. The experimental results showed that the freezing method (i.e., QF and SF) affected the physical properties (moisture content, water activity, and color) of dried mangosteen pericarp. The antioxidant activities (DPPH and ABTS) of the SF + HAD scheme (28.20 and 26.86 mg Trolox/g DW of mangosteen pericarp) were lower than the SF + FD scheme (40.68 and 41.20 mg Trolox/g DW of mangosteen pericarp). The α-mangostin contents were 82.3 and 78.9 mg/g DW of mangosteen pericarp for FD and HAD, respectively; and the corresponding TPC were 1065.57 and 783.24 mg GAE/g DW of mangosteen pericarp. The results of this study suggest that the drying process had a negligible effect on bioactive compounds. Essentially, the SF + HAD technology is the most operationally and economically viable scheme to process mangosteen pericarp.

## 1. Introduction

**Figure 1:**
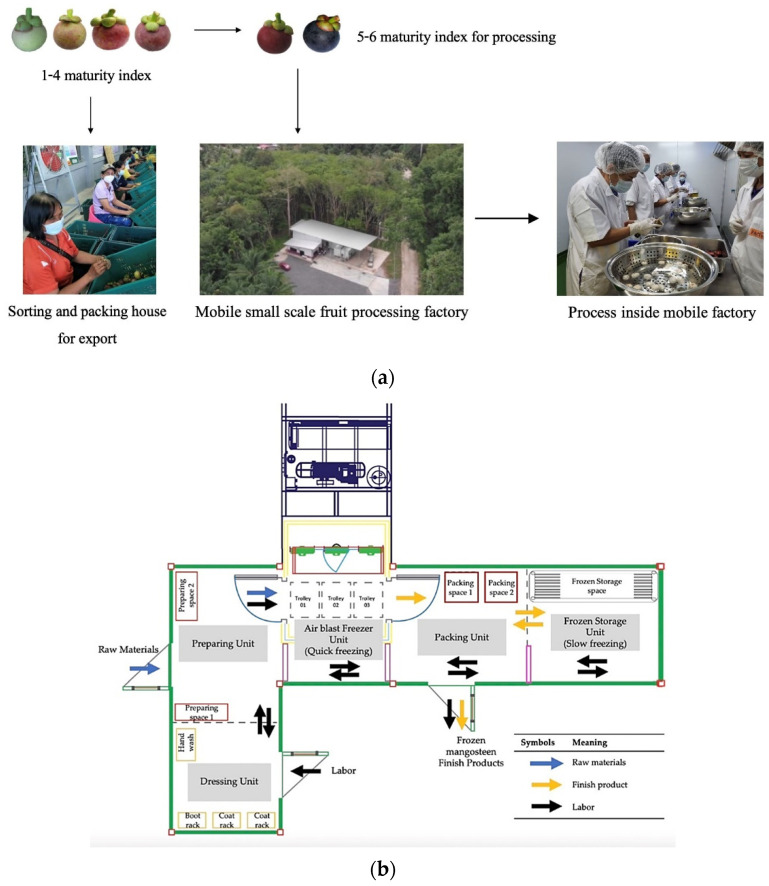
The existing mobile food processing factory under the zero–waste concept for a small– scale operation for processing of mangosteen products at the community enterprise of Lang Suan, Chumphon province, Thailand: (**a**) Processing of mangosteen in a mobile factory, (**b**) layout of the mobile food factory.

Chumphon province is located in the south of Thailand. The province’s largest mangosteen growing area is in *Lang Suan* district (latitude 9°56′42″ N and longitude 99°4′42″ E), with a total cultivation area of 935.0 km^2^ and an estimated production of 4484 tons/year. Mangosteen fruits are harvested during August–September. After harvesting, the fruits are sorted into six grades according to the ripening index (Figure 1a) [1]. The fruits at the third stage of ripening index are destined for export, and those at the fourth to sixth stages (ripe to fully ripe) are sold locally or processed for mangosteen flesh [2]. After processing, the mangosteen pericarp is disposed of in landfills and/or burned in the open, causing land and air pollution.

The existing post-harvest processing system consists of the preparation unit, air blast freezer unit, packing unit, and cold storage unit (Figure 1b). The post-harvest processing system was converted from four shipping containers and retrofitted with freezers and instruments to process mangosteen fruits for mangosteen flesh. Each of the processing units can be hauled by a trailer truck to remote plantations, thereby increasing productivity while saving on transportation cost and time [3,4,5,6]. The post-harvest processing system can be deployed to tackle excess supply of mangosteen fruits, especially during the harvest months. The total capacity of the existing post-harvest processing system is 3000 kg of mangosteen fruit/day.

In mangosteen fruit processing, pericarp and calyx (i.e., biowaste) account for 50.34% and 11.9% of mangosteen fruit, respectively, while the mangosteen flesh accounts for 37.76% [7,8]. In the literature, mangosteen pericarp have been evaluated for their chemical compositions, and the main bioactive compounds in mangosteen pericarp are xanthones, consisting mainly of α- and γ-mangostin [6,9,10,11]. Xanthones have been reported to be useful in various pharmaceutical products for chemopreventive or therapeutic functions [12,13,14,15,16,17,18]. Hence, the recovery of bioactive compounds from byproducts of the mangosteen process is one of our research goals, especially when it comes to finding a process that is sustainable and cost-effective.

The processes of drying and freezing are highly effective and economically viable for enhancing the shelf life of stored products. They can improve cellular structures and alter the porosity of cell walls or membranes, which may increase the extraction efficiency of bioactive compounds [19,20,21]. Moreover, the change in microstructure reduces the drying time [22]. As such, freezing or drying have been widely adopted in numerous industrial applications and reported in scientific research activities [23,24,25]. In principle, freezing can change the structure of plant tissues due to the formation of ice crystals [26], while drying reduces or removes water from the plant material [27]. It inhibits metabolic processes, which minimizes changes in the plant’s chemical composition [28]. Previous studies have shown that the HAD process was used to preserve mangosteen pericarp that yielded 31–61 mg/g DW of α-mangostin [29,30,31].

Depending on the processing condition, we hypothesized that it was possible to obtain a higher level of α-mangostin from mangosteen pericarp at an industrial scale. To our knowledge, there has not been any publications reporting effective recovery of α-mangostin from mangosteen pericarp, a byproduct collected from the mobile processing system; hence, this was the main objective of this study. Two sequential processing steps (freezing then drying) were proposed to be added to the existing frozen mangosteen mobile processing unit. We mainly compared yield and antioxidant activities of α-mangostin extracts from two freezing units: quick freezing (QF) and slow freezing (SF), and two drying processes: hot air drying (HAD) and freeze-drying (FD). The information obtained from this study can be used to demonstrate upcycling of mangosteen pericarp into a value-added bioactive compound. This can also be applicable to other produce wastes, and certainly supports sustainable agriculture and environment.

## 2. Materials and Methods

### 2.1. Raw Materials and Sample Preparation

Ripe mangosteen (*Garcinia mangostana* L.) fruits (the 6th stage; dark purple to black with little or no red color remaining on the peel; Figure 1a) were obtained from the community enterprise of Lang Suan, Chumphon province, Thailand, operating with verified GAP (Good Agricultural Practices) and transported to King Mongkut’s Institute of Technology Ladkrabang (KMITL), Bangkok, within 48 h after harvest. Fruits were washed with peroxyacetic acid (0.04%) and peeled, and the pericarps were separated. The pericarps were coarsely ground using a blending machine (CAMP, Bangkok, Thailand). The initial moisture content of fresh mangosteen pericarp was 61.16 ± 0.36%wb. Ground mangosteen pericarp samples were then packed in linear low-density polyethylene (LLDPE) bags (size 25.4 × 38.1 cm) and kept at −20 °C until further use.

### 2.2. Freezing and Drying Process Description

**Figure 2:**
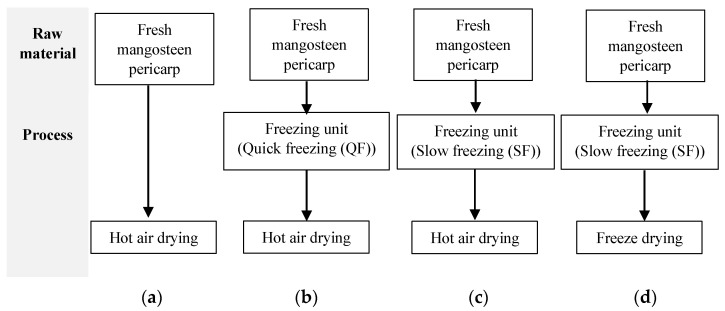
Comparisons of different freezing units and drying processes of dried mangosteen pericarps: (**a**) Hot air drying (HAD; control); (**b**) QF + hot air drying (HAD); (**c**) SF + hot air drying (HAD); and (**d**) SF + freeze-drying (FD).

Before drying, ground mangosteen pericarp samples were divided into two parts for two different freezing treatments: QF and SF. QF was carried out in the air blast freezer equipped with a semicontact plate freezer (R404a: non-CFC, Compact freeze, PATKOL, Bangkok, Thailand) operated at −38 °C with cool air velocity of 3 m/s for 2 h. SF was performed using a mobile cold room (Cool Innotech, Bangkok, Thailand) operated at −25 °C for 2 weeks. For comparison purposes, a fresh sample without freezing was used as a control. Samples were independently prepared in triplicate. All frozen ground mangosteen pericarp samples were thawed at 4 °C for 12 h until the final temperature was 4 °C. After thawing, QF, SF, and fresh mangosteen pericarp samples were separately placed on aluminum trays and dried at 60 °C with an air velocity 1.3 m/s for 7 h using HAD [32]. The drying was carried out until the moisture content was between 2 and 10%, which is the range recommended for dried food production [33]. The dried mangosteen pericarp was then ground by hammer mill (HM−030C, Unique Tools, Chachoengsao, Thailand) and sieved through a 60 mesh (250 µm) sieve. The ground dried mangosteen pericarp was retained in sealed aluminum bags (17.78 × 27.94 cm) and stored at room temperature (25 °C) until further analysis (Figure 2a–c).

Freeze-drying was carried out by placing ground SF mangosteen pericarp on aluminum trays, and the Kryo “D” Freezer machine was used to freeze and vacuum-dry the product (I.T.C, Bangkok, Thailand). In the FD process, the ground mangosteen pericarp underwent three stages: freezing, primary drying, and secondary drying under a vacuum pressure of <40 Pa (Figure 2d). The ground mangosteen pericarp was frozen at −50 °C in an air blast freezer before placing in a semicontact plate freezer at −20 °C (i.e., the primary drying temperature). The secondary drying phase was carried out at 50 °C until the core temperature of the sample (49–50 °C) was different from shelf temperature by 1 °C. In the experiment, to measure the core temperature, the coarsely ground mangosteen pericarp was packed tightly in rectangular-shaped polyethylene (PE) plastic bags. Prior to freeze-drying, the plastic bag was removed and a temperature probe was inserted through the rectangular block of the ground mangosteen pericarp around its center. The temperature probe was left inside for 48 h (i.e., the FD duration). The FD process was terminated after 90 h of drying. The dried pericarp was subsequently ground and sieved through a 60-mesh sieve.

### 2.3. Analytical Methods

#### 2.3.1. Drying Rate

The drying curve plots of the moisture content (MC; kg water/kg sample, dry basis) versus time of drying rate (DR; kg/kg.min^−1^, dry basis) were calculated using Equation (1) as per Pongsuttiyakorn et al. [32].

R = (W_s_/A)(dM/dt) (1)

where R is the drying or evaporation rate (kg/m^2^h), A is the surface area of evaporation (m^2^), W_s_ is the solid weight of the sample (kg), and dM/dt is the mass of water that evaporates per unit time (kg/h). In this study, A is the area of an aluminum tray (27 cm × 37 cm (W × L) equivalent to 0.27 m × 0.37 m, so the surface area of evaporation is 0.999 m^2^.

#### 2.3.2. Moisture Content

The moisture content of dried mangosteen pericarp samples was determined in triplicate using an HAD method; 3.0 g of samples was dried in an hot air oven (UF55, Memmert GmbH, Schwabach, Germany) at 105 ± 5 °C until a constant weight was obtained, as per Raja et al.; AOAC [25,34]. Moisture content was expressed as percentage (wet basis) and calculated using the following equation (Equation (2)).

Moisture content = [(W_1_ − W_2_)/W_1_] × 100 (2)

where W_1_ is the sample weight before drying (g), and W_2_ is the sample weight after drying (g).

#### 2.3.3. Water Activity

Dried mangosteen pericarp (3 g) was taken to determine water activity using the electronic water activity meter (Decagon Model, AquaLab, Washington, DC, USA) following Zhang et al. [35]. Three replications were performed for each sample.

#### 2.3.4. Color

The color of dried mangosteen pericarp was measured in triplicate using the method of Raja et al. [25] by a spectrophotometer (ColorFlex, Hunter Lab, Reston, VA, USA) in terms of CIE L*, a*, and b* values. L* denotes the lightness of the sample ranging from 0 (black) to 100 (white), a* indicates greenness (−) or redness (+), and b* represents blueness (−) and yellowness (+).

#### 2.3.5. Microstructural Analyses

The morphological properties were observed by scanning electron microscopy (SEM) of the dried mangosteen pericarp samples following Xiao et al. [36]. For SEM observation, the sample was mounted on a metal stub, then coated with gold. Samples were observed using a scanning electron microscope and energy-dispersive X-ray spectrometer (JEOL JSM-IT300, Tokyo, Japan), accelerated at 30 kV, with a vacuum pressure of 10 to 650 Pa.

#### 2.3.6. Pore Property Analyses

The pore properties were determined, following Condon [37], by using the N_2_ adsorption isotherms with surface area measurement to characterize porous materials (Quantachrome Autosorb-iQ-C-XR, Anton Paar QuantaTec Inc., Boynton Beach, FL, USA). The specific surface area was obtained from the adsorption isotherms, using the BET theory. Absorption N_2_ gas at 0.95–0.98 of the relative pressure was used to determine the pore volume and pore size.

### 2.4. Quantification of Bioactive Compounds and Antioxidant Activities

#### 2.4.1. Xanthone Extraction and α-Mangostin Assay

Extraction of xanthone was carried out using macerate extraction method, following Yoswathana [29] with minor modifications. Ground dried mangosteen pericarp (5.0 g) was mixed with 50 mL of 60% (*v*/*v*) ethanol mixture, vortexed for 1 min, and macerated overnight (24 h). The solution was centrifuged (Mikro 220R, Hettich, Kirchlengern, Germany) at 4000× *g* for 5 min. The supernatant was collected and filtered through a 0.45 μm syringe filter nylon membrane (Cnw Technologies, Shanghai, China). The extract solution was evaluated for TPC, antioxidant activities, and α-mangostin content by HPLC.

The amount of α-mangostin (mg/g DW of mangosteen pericarp) was assayed by HPLC at 232 nm (Shimadzu, Kyoto, Japan) (column: Inertril ODS-3, 250 × 4.6 mm, 5 μm particle size, GL Science, Tsukuba, Japan). The mobile phase was methanol at isocratic elution at a flow rate of 1 mL/min, the column temperature was maintained at 40 °C, and 1 μL of sample was injected. The aqueous solubility of xanthone was compared with a control, which was α-mangostin of 97% purity (Toronto research chemicals, Nanterre, France).

#### 2.4.2. Determination of Total Phenolic Compounds (TPC)

TPCs were estimated by the Folin–Ciocalteu method [38]. Samples for this assay were the extracted xanthone samples described in Section 2.4.1, mixed with 20 µL of extract, distilled water, and 100 µL of the Folin–Ciocalteu reagent by vortex. After 30 min, 300 µL of 1 N Na_2_CO_3_ was added to the mixture. Absorbance was determined at 725 nm using the UV–visible spectrophotometer (UV-3100PC, VWR International, Shanghai, China). TPC was analyzed and the absorbance was compared with the standard curve of gallic acid. The values were multiplied by dilutions and converted into per gram of ground dried pericarp. The results were reported as mg of gallic acid per g dry weight of mangosteen pericarp (mg GAE/g DW of mangosteen pericarp).

#### 2.4.3. DPPH Radical Scavenging Activity

The antioxidant activity was determined by DPPH radical scavenging assay, following Brand-Williams et al. [39], with minor modifications. The xanthone extract sample was reacted with 2900 µL of DPPH solution for 30 min in the dark. The stock solution was prepared by dissolving 0.1 mM DPPH in methanol. The absorbance was then taken at 515 nm using the UV–visible spectrophotometer (UV-3100PC, VWR International, China). The standard curve was prepared by using concentrations of Trolox. The antioxidant activity was analyzed and the absorbance was compared with the standard curve. The values were multiplied by dilutions and converted into per gram of ground dried pericarp, and results were expressed as mg Trolox per g dry weight of mangosteen pericarp (mg Trolox/g DW of mangosteen pericarp).

#### 2.4.4. ABTS Radical Scavenging Activity

The ABTS radical scavenging activity was determined following the method described in Arnao et al. [40]. ABTS stock solution (7 mM ABTS in 2.45 mM potassium persulfate) was prepared and retained at −20 °C in the dark. The working ABTS solution with an absorbance of 0.68–0.72 at 734 nm was prepared by diluting the stock solution with distilled water. The xanthone sample was mixed with 2980 µL of the ABTS reagent and incubated for 10 min in the dark. Absorbance was measured at 734 nm by the UV–visible spectrophotometer (UV-3100PC, VWR International, China). The antioxidant activity was analyzed and the absorbance was compared with the standard curve. The values were multiplied by dilutions and converted into per gram of ground dried pericarp, and results were expressed as mg Trolox per g dry weight of mangosteen pericarp (mg Trolox/g DW of mangosteen pericarp).

#### 2.4.5. Ferric Reducing Antioxidant Potential (FRAP) Assay

The FRAP assay was performed according to the method described in Benzie and Strain [41]. The ethanolic extract was added to a test tube with 2980 µL of FRAP reagent (pH 3.6). The mixture was incubated for 30 min in a dark room. The absorbance of the solution (blue color) was measured against the acetate buffer (the blank) at 593 nm using the UV–visible spectrophotometer (UV-3100PC, VWR International, China). A standard curve was prepared using concentrations of Trolox. The antioxidant activity was analyzed and the absorbance was compared with the standard curve. The values were multiplied by dilutions and converted into per gram of ground dried pericarp, and results were expressed as mg Trolox per g dry weight of mangosteen pericarp (mg Trolox/g DW of mangosteen pericarp.

### 2.5. Statistical Analyses

The experiments were performed in triplicate using a completely randomized design (CRD). All data are expressed as the mean ± standard deviation (SD). Data analyses were carried out using the IBM SPSS Statistics for Windows, Version 26.0 (IBM Corp., New York, NY, USA). Analysis of variance (ANOVA) and multiple comparisons of means (Tukey’s HSD test) were performed at a 0.05 level of significance.

## 3. Results and Discussion

### 3.1. Effects of Different Freezing and Hot Air Drying Processes on Qualities of Ground Mangosteen Pericarps

The impact of freezing methods on the quality indicators of mangosteen pericarp product was studied. Fresh ground mangosteen pericarp (control) and samples that were previously subject to QF or SF were compared after HAD. The moisture content of fresh mangosteen pericarp and frozen mangosteen pericarp after thawing were 61.16 ± 0.36%wb and 63.27 ± 0.12%wb, respectively.

#### 3.1.1. Drying Kinetics

**Figure 3:**
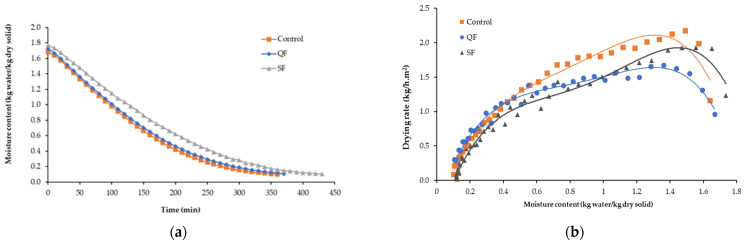
Effects of different freezing methods on drying kinetics during drying of ground mangosteen pericarps. QF: quick freezing; SF: slow freezing. (**a**) Changes in moisture content with time during the drying process. (**b**) Drying rate during the hot air drying (HAD).

Drying curves of the QF, SF, and fresh mangosteen pericarp samples were plotted between moisture content and drying time, as presented in Figure 3a. To evaluate the drying period in this study, we fitted the moisture content data from the initial measurement to a moisture content ratio of 0.1. This is a standard for dried food with a moisture content of the product, not to exceed 10% by weight [32]. The time for the fresh sample to reach a final water content of 0.10 ± 0.06 kg H_2_O/kg (db) was 350 min. Compared with the control, the QF required an additional 20 min to reach the final moisture content (i.e., after 370 min), and the SF reached the same final moisture content 80 min later (i.e., after 430 min). The drying rate was delayed by all the freezing methods.

Generally, the drying rate decreases with time. As the drying temperature decreases, the relative humidity increases, resulting in a lower mass transfer, lower moisture content, and lower drying rate. A characteristic drying curve is presented in Figure 3b. The initial moisture content after thawing of all frozen samples of mangosteen pericarp was the factor influencing the drying rate. The initial moisture content of mangosteen pericarp from all frozen samples was higher than that of the control. The drying rates of the control and QF increased rapidly until the moisture loss stabilized after about 6 h of drying and approximately 7 h of drying for SF. The increase in drying rate of QF and SF could be attributed to the destruction of cell membrane surface [42] and resistance to internal moisture diffusion as a result of physical damage [43]. Freezing of vegetables and fruits disrupted plant cells due to the formation of ice crystals. Water in ice state took up 9% more volume than liquid water, resulting in volume changes or expansion. The degree of freezing-induced expansion depended on several factors, including moisture content, plant cell configuration, solute concentration, freezing temperature, and formation of crystals [26]. Freezing also altered the internal structure of the material. The formation of intracellular and extracellular small ice crystals in QF increased heat and mass transfer rates and decreased water migration, thereby promoting higher drying rates. Meanwhile, large ice crystals formed in cell spaces caused damage to the structure of the SF sample [42,43].

The polynomial models, along with coefficients of determination and the root mean square errors, of QF, SF, and fresh mangosteen pericarps subject to HAD at 60 °C are presented in Table 1. For the control, QF + HAD, and SF + HAD schemes, R^2^ values were 0.9837, 0.9454, and 0.9145, respectively. The R^2^ values for the page, sigmoid, and polynomial models greater than 0.90 indicate a good fit. In this study, the sigmoid and polynomial models gave the highest R^2^ values for the QF + HAD and SF + HAD schemes. The solid line in Figure 3b represents the polynomial model fitted to the actual experimental data. The averaged drying rate was greater at the beginning of the drying process due to evaporation and the moisture from the surface of ground mangosteen pericarp, which later declined with decreasing moisture content, regardless of the freezing method applied. In addition, the normal falling-rate period in the absence of a constant drying rate period observed for the drying curves (Figure 3b) indicated that the internal mass transfer occurred by diffusion. The accelerated drying rate was presumed to be attributed to internal heat generation [44]. Moisture diffusion is the main physical mechanism governing moisture movement during the drying process. A drying rate decreases continuously with improving drying time [45].

#### 3.1.2. Physicochemical Properties

The QF + HAD and SF + HAD schemes had significantly (*p* < 0.05) lower moisture content and water activity (a_w_) than the control (only HAD), as shown in Table 2. The moisture content and water activity of these two samples followed the Thailand standard of dried herbs [46], that is, less than 10% by weight and 0.60, respectively. Water activity involves measuring the degree to which the water within a specific product is not bound and is readily available for physiological reactions and the growth of microorganisms. It is the ratio of water vapor pressure of the product to the vapor pressure of pure water at the same temperature [32], and is expressed on a scale ranging from 0 (the absence of energy) to 1 (an energy level equivalent to that of pure water). On the other hand, moisture content (% by weight) refers to the amount of water in a product, which impacts the physical properties of a substance, such as weight, density, viscosity, conductivity, and others [47]. In HAD, the movement of moisture within solid foods is facilitated by processes such as liquid or vapor diffusion, surface diffusion, hydrostatic pressure differences, or a combination thereof [48]. Moisture content and water activity are interrelated factors that influence the extraction of bioactive compounds from dried mangosteen pericarp due to increased surface and porosity. Neelapong et al. [49] reported that a high moisture content on fresh mangosteen pericarp may interfere with some solvents during extraction. Hence, the lower the moisture content and water activity of dried mangosteen pericarp, the more efficient the extraction.

Color profiles of the ground dried mangosteen pericarp samples were affected by the freezing methods. SF prior to HAD did not affect the color lightness (L*) values of the HAD. However, the freezing methods, both QF and SF, prior to HAD, resulted in samples with lower a* (less redness) and b* (less yellowness) values; based on visual observation, the color of these two samples changed from dark purple (anthocyanin) to brown. This could be attributed to the fact that freezing causes physical changes in food due to ice crystal formation [50]. During freezing, followed by the thawing, the cellular structure is affected by the size and the quantity of ice crystals. QF led to the formation of smaller ice crystals in the intracellular space, while SF induced the formation of larger ice crystals. Both freezing methods likely led to textural damage of the cell wall, middle lamella, and protoplast, thus leading to less pigment anthocyanin [51,52]. Moreover, the effect of pigment loss could be attributed to the prolonged thawing period (i.e., at 4 °C for 12 h until the final temperature was 4 °C) used in this study. Pigment losses during thawing could be due to enzymatic degradation of anthocyanins by polyphenol oxidase (PPO), and peroxidase (POD) activities are still active at lower temperatures. Holzwarth et al. [53] reported the highest pigment losses (21.4%) when strawberries were thawed at 4 °C for 24 h.

The geometric characteristics of size, shape, volume, surface area, and porosity are important for a process design for handling and processing operations of many food materials. It is also necessary to use these properties for determination of suitable product for further specified use. Results from Table 3 show the effects of different freezing (QF and SF) methods combined with HAD (at 60 °C) processes on the surface area, pore volume, and pore size of ground dried mangosteen pericarps. The QF + HAD had the largest surface area (80.75 m^2^/g), which was twice as much as that of the control (HAD; 40.16 m^2^/g), whereas the surface area of SF + HAD was the smallest (29.61 m^2^/g). These surface area values corresponded to the pore volumes of 0.137, 0.070, and 0.054 cm^3^/g, respectively (Table 3). Pore volume is the volume of the void or air inside a material [54]. The pore size of SF + HAD was larger (4.850 nm) as compared with the QF + HAD (4.174 nm). Pore sizes between 2 and 50 nm are mesopores, and above 50 nm are macropores [55]. The average pore size of ground dried mangosteen pericarp was approximately 4.8 nm (i.e., within the mesopore range). The results corresponded to those reported by Levin et al. [56], where SF had big ice crystal formation, causing a collapse of pore structures during drying. Tamer et al. [57] reported that air temperature for a drying process had no significant impact on the final shrinkage and porosity values. During drying, the porosity of the samples first increased until a critical value, at which point further decrease in moisture resulted in collapse of pores. The high adsorption efficiency of mangosteen pericarp could be attributed to large surface area and micropore structure [58]. In Table 2, although the phytochemical extraction efficiency of QF + HAD scheme was higher than SF + HAD, the differences were statistically insignificant. In Table 3, the QF + HAD scheme exhibited the largest surface area and highest total pore volume, while the SF + HAD scheme had the largest pore size. Essentially, the surface area and micropore structure affected the extraction efficiency of bioactive compounds because an increase in contact surface area with the solvent led to an increase in mass transfer of exposed soluble matter [11,25].

**Figure 4:**
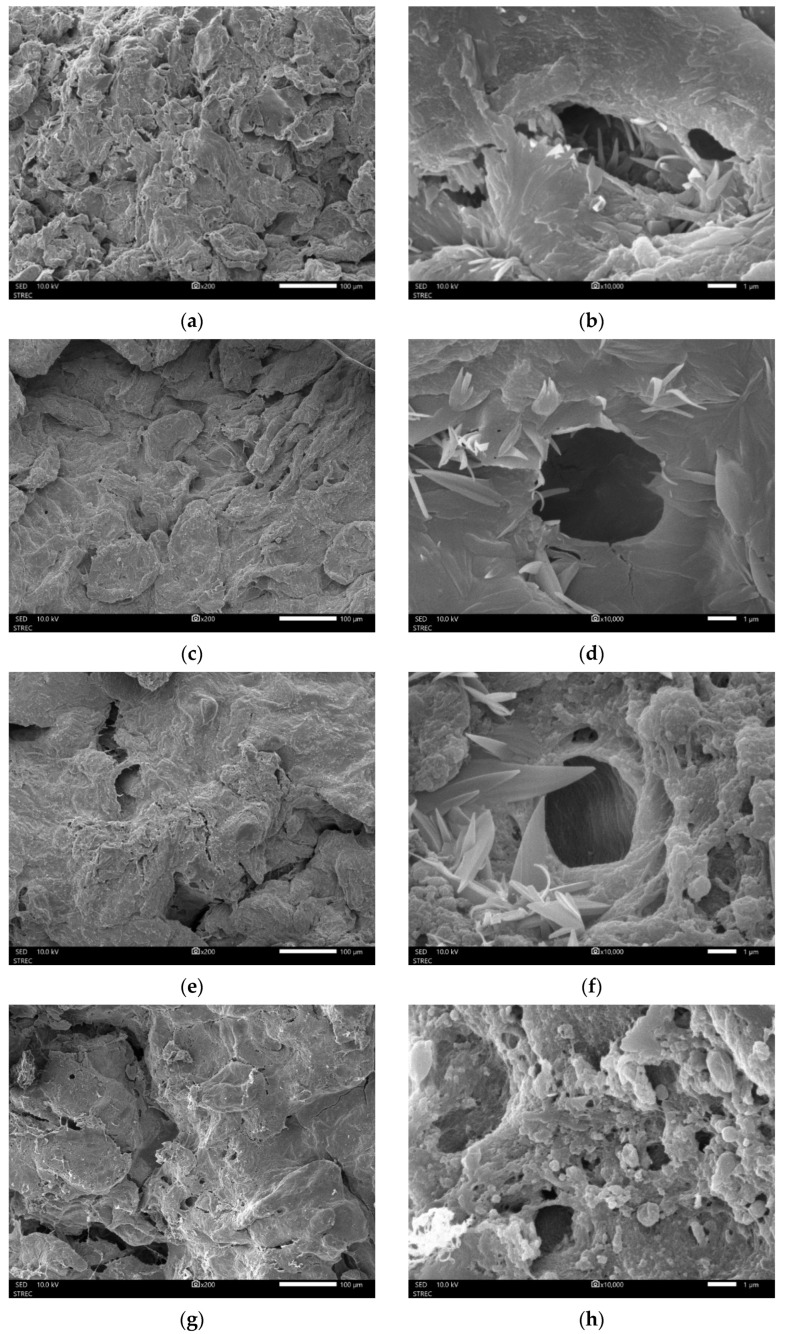
×200 (the left side) and ×10,000 (the right side) SEM micrographs of mangosteen pericarp samples, respectively. (**a**,**b**) HAD (hot air drying). (**c**,**d**) Quick freezing + HAD. (**e**,**f**) Slow freezing + HAD. (**g**,**h**) Slow freezing + freeze-drying.

Figure 4 shows SEM micrographs of the ground dried mangosteen pericarp matrix obtained by QF and SF and HAD. The quality differences in pore size, tortuosity, and surface area appearance were observed as a result of freezing methods. Prior freezing process generated bigger size pores than the control (only HAD). The surface area of the SF + HAD had irregular surface around the pores, whereas that of QF + HAD was smoother. From the results of SEM micrographs, it was obvious that freezing changed the microstructure and the surface area and pore size [55]. Thus, QF + HAD increased surface area, whereas SF + HAD increased pore size.

#### 3.1.3. Bioactive Compounds

Mangosteen pericarp contains a large amount of xanthone (69.02%) [29], an organic compound with several medicinal benefits. In this study, both freezing methods (QF and SF) achieved a significantly higher α-mangostin content (78.88–84.16 mg/g DW of mangosteen pericarp) as compared to the control (HAD only; 66.94 mg/g DW of mangosteen pericarp). Meanwhile, the α-mangostin content of QF + HAD and SF + HAD schemes were 25.72% and 17.83% higher than the control. Smaller particle size increases the extraction efficiency of bioactive compounds in pomegranate peel extraction due to a larger surface area per mass unit [59], consistent with the QF + HAD result of this research. In SF + HAD, large ice crystals formed during the freezing process might cause damage to insoluble constituents binding xanthone [60], while the drying process could affect the porosity, bulk and particle density, and shrinkage [25]. However, these undesirable phenomena improved the extractability of bioactive compounds.

The TPC content of QF + HAD and SF + HAD was 750.45 mg GAE/g DW of mangosteen pericarp and 783.24 mg GAE/g DW of mangosteen pericarp, respectively, which were lower than that (792.34 mg GAE/g DW of mangosteen pericarp) of the control (HAD only). Vallespir et al. [61] reported that a TPC content loss was higher for the sample undergoing freezing pretreatment before drying [61]. The growth of ice crystals may break the cellular walls and promote bioactive compound losses, and HAD further caused intensive oxidation due to a long heat exposure period [62]. Antioxidant assays in this study, including the 2,2 diphenyl-1-picryl hydrazyl (DPPH) and the 2,2-azino-bis (3-ethylbenzothiazoline-6-sulphonic acid, ABTS) assays, were used to determine the radical scavenging ability, while the ferric reducing antioxidant power (FRAP) assay was used to measure the reducing power of the dried mangosteen pericarps (Table 2). The results showed that both freezing pretreatments prior to HAD did not significantly influence antioxidant activities of dried ground mangosteen pericarp. However, the SF + HAD sample tentatively had lower (but not significantly) DPPH and FRAP values compared to the control; this could be due to ice crystals generated during slow freezing that caused cell damage [63], and the PPO enzyme responsible for catalyzation due to the oxidation of phenolic compounds using molecular oxygen [64]. Decreases in antioxidants and their antioxidant activities in fruits are due to some handling practices such as chilling injury, freezing, irradiation, incorrect holding temperature and relative humidity, and other factors such as stress and fungal decay [10,24,64]. Overall, the plant extracts prepared from frozen material likely had less antioxidant potential and polyphenol content, including anthocyanins and flavonoids.

### 3.2. Effects of Slow Freezing Unit and Different Drying Processes on Qualities of Ground Mangosteen Pericarps

In this section, the parameters of the SF pretreatment are the same for the subsequent HAD (at 60 °C) or FD (at −20 °C then at 50 °C). FD is widely used because it yields a high quality of biological materials and pharmaceuticals [12,13,14,15,16,17,18].

#### 3.2.1. Physicochemical Properties

The moisture content and water activity were major quality parameters of dried products. According to Table 2, the SF + FD had significantly (*p* < 0.05) lower moisture content (2.42%) and water activity (0.13) than those of the SF + HAD (4.60% and 0.34, respectively). Water activity is often the most important factor to control the growth of spoilage and pathogenic microorganisms. Dry food should have a water activity below 0.6 to have extended shelf life.

The color parameters of ground dried mangosteen pericarp were significantly (*p* < 0.05) affected by different drying processes. The SF + FD showed higher L* and a* values (lighter and more reddish) than SF + HAD (Table 2), and the b* value (less yellow) was significantly lower. The FD retained color of products by minimizing color loss occurring due to thermal, oxidative, or light degradation [65]. The purple color matter of the mangosteen fruit pericarp mostly contains anthocyanins. Changes of anthocyanin are dependent on light, temperature, pH, metal ions, enzymes, and oxygen [2]. Çoklar et al. [66] reported that oven-drying and sun-drying methods led to more than 95% anthocyanin reduction in grape. Moreover, anthocyanins belong to the phenolic group that exhibits antioxidant properties, hence the higher antioxidant activities (DPPH and ABTS, Table 2).

Different drying processes affected pore properties of ground dried mangosteen pericarps (Table 3). The surface area of the SF + FD was smaller (20.93 m^2^/g) than that of the SF + HAD (29.61 m^2^/g). In contrast, the pore size of the SF + FD was larger (7.274 nm) compared with that of the SF + HAD (4.850 nm). Porosity and tortuosity of the material are used to calculate the effective diffusivity during mass transfer processes [54]. In an extraction process, particle size plays an important role in controlling the yield of an extract [67]. The surface area increases with decreasing particle size. The surface area also increases if the particle has smaller pores. Thus, the surface area of the material in contact with the solvent is increased due to the mass transfer rate, and a higher yield of extract can be obtained [67,68]. In this case, the surface area and pore size of ground dried mangosteen pericarp affected the extraction of bioactive compounds, as shown in Table 2. The SEM micrographs could clearly demonstrate the different surface structures of the ground dried mangosteen pericarp due to different drying processes. The SF + FD had irregular surfaces around the pores (Figure 4g,h), which was similar to that of SF + HAD (Figure 4e,f).

#### 3.2.2. Bioactive Compounds

The α-mangostin and TPC content (82.30 and 1065.57 mg GAE/g DW of mangosteen pericarp) of the SF + FD were insignificantly higher than the SF + HAD (78.88 and 783.24 mg GAE/g DW of mangosteen pericarp) (Table 2). However, the antioxidant activities (DPPH and ABTS) of the SF + FD were higher than the SF + HAD (40.68–41.20 vs. 26.86–28.20 mg Trolox/g DW of mangosteen pericarp). The FRAP value of the SF + FD was insignificantly higher than that of the SF + HAD. The findings indicated that the drying process (FD and HAD) had a negligible effect on the content of α-mangostin. Nevertheless, the α-mangostin content of SF + HAD (78.88 mg/g DW of mangosteen pericarp) was significantly higher when compared to previous studies that used only HAD to process mangosteen pericarp (31–61 mg/g DW of mangosteen pericarp) [29,69].

The bioactive compounds being studied were TPC and *α*-mangostin, and the results showed that they were insignificantly different between SF + HAD and SF + FD. However, the higher antioxidant activities (DPPH and ABTS) of FD could be attributed to higher content of anthocyanins, as evidenced by the purple color of the coarsely ground mangosteen pericarp [70,71]. Meanwhile, the color of ground mangosteen pericarp of HAD was dark brown, suggesting lower anthocyanins in the processed pericarp (note: anthocyanins are a group of antioxidants found in red, blue, and purple fruits and veggies). Xanthones belong to a class of polyphenolic compounds mainly found in mangosteen pericarp, and have been widely reported for their antioxidant activity [6,70,71,72,73,74,75]. Suvarnakuta et al. [76] reported the decrease in xanthone in mangosteen pericarp due to HAD when the drying temperature was more than 75 °C. Therefore, thermal factors could be critical for the losses of xanthones during drying [30,32,76].

### 3.3. Potential Economic Advantage of Recovered α-Mangostin

Despite having a low economic value (USD 0.2/kg), the fresh mangosteen pericarp can be upcycled into a high-value product by extracting the bioactive compound, specifically α-mangostin (USD 1.5/g) [31]. Our results showed that 1 kg of dried mangosteen pericarp could contain 77.88 g of α-mangostin in the crude extract (Table 2), which was 15.7 g pure form of α-mangostin, and the price was increased by 15 times that of the fresh mangosteen pericarp, a waste material from the frozen mangosteen production. Dried mangosteen pericarp has four basic cost components, including raw material cost, labor cost, packaging cost, and operation cost (freezing and drying process), to calculate per 1 kg of production cost. In all processing schemes, there are raw material costs, packaging costs, and labor management costs, with the following values: USD 0.58, 0.075, and 0.074/kg, respectively. The operating costs were higher for all processing schemes due to association with electricity use for freezing and drying process. The mangosteen pericarp processing schemes involved four major processes: pericarp separation, coarse grinding, freezing (QF or SF), and drying (HAD or FD). The electricity consumption and cost to grind mangosteen pericarp were roughly the same for all pericarp processing schemes. However, the electricity consumption of FD was much higher than HAD. FD utilizes a power rating of 54 kilowatts, vis à vis 5.71 kilowatts for HAD. In addition, the drying duration of FD (48 h) was considerably longer than HAD (7 h). The QF + FD scheme was excluded from this study for the following reasons: (i) After the QF process but prior to FD, the coarsely ground mangosteen pericarp requires refrigeration to maintain the product quality. This requirement unnecessarily increases the overall cost of the QF + FD scheme; (ii) FD consumes a large amount of electricity, resulting in high electricity cost; (iii) In practice, FD requires expertise to operate and maintain. These reasons render the QF + FD scheme operationally (due to the third reason) and economically (due to the first and second reasons) unattractive. The operation costs of HAD (control), SF + HAD, and QF + HAD schemes were USD 0.56, 1.05 and 1.18/kg of ground dried mangosteen pericarp, respectively. Meanwhile, the operation cost of SF + FD was prohibitively expensive (USD 72.43/kg of ground dried mangosteen pericarp), making it not economically viable. In addition to time, HAD requires lower initial investment capital and is easier to operate and maintain, in comparison with FD. Since users of the proposed mangosteen pericarp processing technology are either small-scale farmers with limited financial resources or low-skilled workers without advanced training, HAD is, thus, more operationally and economically favorable.

This indicates that the capital cost of dried mangosteen pericarps produced with SF is simply the convenient handing process. The use of simple machines and easy preparation with no complicated steps results in the ease of labor. The choice of QF vs. SF depends on the manufacturer. Reducing production costs by converting waste materials at the initial stage to a high-quality α-mangostin extract can help increase profitability in the business.

## 4. Conclusions

The initial mangosteen pericarps suitable for the α-mangostin extraction may be subjected to a freezing pretreatment prior to a drying process. Freezing had positive effects towards the physicochemical properties of the mangosteen pericarps such as moisture content, water activity, surface area and pore size, which, in turn, affected the extraction efficiency of the α-mangostin content. Both QF and SF pretreatment prior to HAD achieved a significantly higher α-mangostin content than HAD alone. The α-mangostin contents were 82.3 and 78.9 mg/g DW of mangosteen pericarp for FD and HAD, respectively, and the corresponding TPC were 1065.57 and 783.24 mg GAE/g DW of mangosteen pericarp. The differences in bioactives were statistically insignificant, suggesting that the drying process had a negligible effect on bioactive compounds. Moreover, HAD could be performed in a shorter time (7 h) than freeze-drying (48 h). Therefore, the SF + HAD scheme is the most operationally and economically viable scheme to process mangosteen pericarp. The SF + HAD scheme requires low initial investment capital and is easy to operate and maintain. In addition, since the users of the proposed mangosteen pericarp processing technology are either small-scale farmers with limited financial resources or low-skilled workers without advanced training, the SF + HAD scheme is, thus, an operationally and economically ideal option.

## Figures and Tables

**Table 1:** The polynomial models of different freezing methods combined with the hot air drying process for ground mangosteen pericarp.

Methods	Model	R	$R^{2}$
Control	$y=-2.8675x^{4}+9.3596x^{3}-11.347{0x}^{2}+7.2240x-0.4840$	0.8971	0.9837
QF + HAD	$y=-2.5333x^{4}+9.1444x^{3}-11.9360x^{2}+7.1969x-0.3752$	0.8890	0.9454
SF + HAD	$y=-4.6792x^{4}+15.9950x^{3}-18.0200x^{2}+8.7914x-0.6923$	0.8371	0.9145

Control: hot air dry (HAD) without freezing; QF; quick freezing, SF; slow freezing, R; RMSE.

**Table 2:** Physicochemical properties, bioactive compounds, and antioxidant activities of ground dried mangosteen pericarp as affected by different freezing methods combined with different drying processes.

	Process			
	Control	QF + HAD	SF + HAD	SF + Freeze-Drying
Physical properties				
Moisture content (%)	9.89 ± 0.21 ^d^	6.16 ± 0.18 ^c^	4.60 ± 0.11 ^b^	2.42 ± 0.16 ^a^
Water activity (a_w_)	0.51 ± 0.01 ^d^	0.43 ± 0.00 ^c^	0.34 ± 0.00 ^b^	0.13 ± 0.00 ^a^
Color				
L*	43.22 ± 0.06 ^b^	40.44 ± 0.12 ^a^	43.29 ± 0.13 ^b^	44.72 ± 0.12 ^c^
a*	19.55 ± 0.02 ^d^	17.73 ± 0.08 ^b^	17.35 ± 0.14 ^a^	18.99 ± 0.06 ^c^
b*	26.23 ± 0.19 ^b^	24.77 ± 0.12 ^a^	25.80 ± 0.22 ^b^	18.20 ± 0.19 ^a^
Bioactive compounds				
α-mangostin (mg/g DW of mangosteen pericarp)	66.94 ± 0.80 ^a^	84.16 ± 0.46 ^b^	78.88 ± 0.74 ^b^	82.30 ± 0.27 ^b^
Total phenolic compounds (mg GAE/g DW of mangosteen pericarp)	792.34 ± 33.23 ^a^	750.45 ± 40.06 ^a^	783.24 ± 49.58 ^a^	1065.57 ± 30.17 ^a^
Antioxidant activity				
DPPH	32.90 ± 1.03 ^ab^	32.05 ± 3.21 ^a^	28.20 ± 0.54 ^a^	40.68 ± 1.41 ^b^
ABTS	25.47 ± 1.13 ^a^	27.18 ± 0.52 ^a^	26.86 ± 0.22 ^a^	41.20 ± 1.17 ^b^
FRAP	0.32 ± 0.07 ^a^	0.27 ± 0.05 ^a^	0.28 ± 0.01 ^a^	0.38 ± 0.07 ^a^

Control: hot air dry (HAD) without freezing; QF: quick freezing; SF: slow freezing. DPPH: 2,2 diphenyl-1-picryl hydrazyl assay (mg Trolox/g DW of mangosteen pericarp), FRAP: ferric reducing antioxidant power assay (mg Trolox/g DW of mangosteen pericarp), ABTS: 2,2-azino-bis (3-ethylbenzothiazoline-6-sulphonic acid assay (mg Trolox/g DW of mangosteen pericarp). Data are expressed as mean ± standard deviation (SD). Means in the same row followed by different letters differ significantly (*p* < 0.05).

**Table 3:** Pore properties of ground dried mangosteen pericarps affected by different freezing methods combined with different drying processes.

Pore Properties	Control (HAD)	QF + HAD	SF + HAD	SF + Freeze-Drying
Specific surface area (m^2^/g) Single point surface area ^a^	40.16 ± 0.26 ^d^	80.75 ± 0.15 ^c^	29.61 ± 0.09 ^b^	20.93 ± 0.04 ^a^
BET surface area ^b^	58.28 ± 0.18 ^c^	131.50 ± 0.20 ^d^	45.28 ± 0.28 ^b^	36.26 ± 0.11 ^a^
Total pore volume (cm^3^/g) ^c^	0.070 ± 0.005 ^b^	0.137 ± 0.003 ^c^	0.054 ± 0.002 ^a^	0.066 ± 0.003 ^b^
Pore size (average pore diameter, nm) ^d^	4.772 ± 0.04 ^b^	4.174 ± 0.07 ^a^	4.850 ± 0.02 ^c^	7.274 ± 0.01 ^d^

^a^ Calculated at a relative pressure of 0.30 using the BET method; ^b^ calculated at a relative pressure range of 0.04–0.15 (4 points) using the BET method; ^c^ calculated at a relative pressure of approximately 0.949 (i.e., saturated adsorption); ^d^ obtained by the ratio of the total pore volume (V) to the BET surface area (SBET) (i.e., average pore diameter = 4 V/SBET), assuming a cylindrical pore geometry. Control: hot air dry (HAD) without freezing; QF: quick freezing; SF: slow freezing.

## Data Availability

The original contributions presented in the study are included in the article, further inquiries can be directed to the corresponding author.

## Abbreviations

ABTS	2,2′-azino-bis (3-ethylbenzthiazoline-6- sulphonic acid)) radical cation decolorization assay
BET	Brunauer-Emmett-Teller surface area analysis
DPPH	1,1-diphenyl-2-picrylhydrazyl radical scavenging activity
FRAP	Ferric reducing antioxidant potential assay
GAE	Gallic acid
HAD	Hot air drying
FD	Freeze-drying
PPO	Polyphenol oxidase
POD	Peroxidase
QF	Quick freezing
QF + HAD	Quick freezing and hot air drying process
SF	Slow freezing
SF + HAD	Slow freezing and hot air drying process
SF + FD	Slow freezing and freeze drying process
SEM	Scanning electron microscope
TPC	Total phenolic content
